# Supplementary material for: Personalized bacteriophage therapy to treat pandrug-resistant spinal Pseudomonas aeruginosa infection
Source: Nat Commun. 2022 Jul 22;13:4239. doi: 10.1038/s41467-022-31837-9 (PMC9306240; doi:10.1038/s41467-022-31837-9)
Supplement: Supplementary file 2 — Description of Additional Supplementary Files [file 41467_2022_31837_MOESM2_ESM.docx]

**Supplementary movie legend**

Movie showing the favorable evolution of the patient during the follow-up (21 months), the latter walking without pain and without clinical signs in favor of a persistent infection.
