# Supplementary material for: Personalized bacteriophage therapy to treat pandrug-resistant spinal Pseudomonas aeruginosa infection
Source: Nat Commun. 2022 Jul 22;13:4239. doi: 10.1038/s41467-022-31837-9 (PMC9306240; doi:10.1038/s41467-022-31837-9)
Supplement: Supplementary file 4 — Reporting Summary [file 41467_2022_31837_MOESM4_ESM.pdf]

nature portfolio

Corresponding author(s): DBPR NCOMMS-22-01270-T

Last updated by author(s): Feb 8, 2022

Reporting Summary

Nature Portfolio wishes to improve the reproducibility of the work that we publish. This form provides structure for consistency and transparency in reporting. For further information on Nature Portfolio policies, see our Editorial Policies and the Editorial Policy Checklist.

Statistics

For all statistical analyses, confirm that the following items are present in the figure legend, table legend, main text, or Methods section.

n/a	Confirmed	
☒	☐	The exact sample size (*n*) for each experimental group/condition, given as a discrete number and unit of measurement
☒	☐	A statement on whether measurements were taken from distinct samples or whether the same sample was measured repeatedly
☒	☐	The statistical test(s) used AND whether they are one- or two-sided<br>*Only common tests should be described solely by name; describe more complex techniques in the Methods section.*
☒	☐	A description of all covariates tested
☒	☐	A description of any assumptions or corrections, such as tests of normality and adjustment for multiple comparisons
☒	☐	A full description of the statistical parameters including central tendency (e.g. means) or other basic estimates (e.g. regression coefficient) AND variation (e.g. standard deviation) or associated estimates of uncertainty (e.g. confidence intervals)
☒	☐	For null hypothesis testing, the test statistic (e.g. *F*, *t*, *r*) with confidence intervals, effect sizes, degrees of freedom and *P* value noted<br>*Give P values as exact values whenever suitable.*
☒	☐	For Bayesian analysis, information on the choice of priors and Markov chain Monte Carlo settings
☒	☐	For hierarchical and complex designs, identification of the appropriate level for tests and full reporting of outcomes
☒	☐	Estimates of effect sizes (e.g. Cohen's *d*, Pearson's *r*), indicating how they were calculated

Our web collection on statistics for biologists contains articles on many of the points above.

Software and code

Policy information about availability of computer code

Data collection	No software was used
Data analysis	PATRIC v3.6.12<br>blastn (https://blast.ncbi.nlm.nih.gov/)<br>PhageAI (https://app.phage.ai/phages/)<br>Virulence Factors Database (VFDB, http://www.mgc.ac.cn/VFs/main.htm)

For manuscripts utilizing custom algorithms or software that are central to the research but not yet described in published literature, software must be made available to editors and reviewers. We strongly encourage code deposition in a community repository (e.g. GitHub). See the Nature Portfolio guidelines for submitting code & software for further information.

Data

Policy information about availability of data

All manuscripts must include a data availability statement. This statement should provide the following information, where applicable:

- Accession codes, unique identifiers, or web links for publicly available datasets
- A description of any restrictions on data availability
- For clinical datasets or third party data, please ensure that the statement adheres to our policy

The full genome sequence and associated data for phage vB_PaeP_4029, vB_PaeP_4032, and vB_PaeP_4034 were deposited in GenBank (https://www.ncbi.nlm.nih.gov/genbank/) under BioProject number PRJNA691459. The genome accession numbers are ON815901, ON815902, and ON815903, respectively. The BioSample numbers are SAMN28810961, SAMN28810962, and SAMN28810963, respectively. The raw reads were deposited under accession numbers SRX15796894, SRX15796895, and SRX15796896, respectively.

The authors declare that all other data supporting the findings of this study are available within the article and its Supplementary Data file. Source data are provided with this paper.

Field-specific reporting

Please select the one below that is the best fit for your research. If you are not sure, read the appropriate sections before making your selection.

[x] Life sciences [ ] Behavioural & social sciences [ ] Ecological, evolutionary & environmental sciences

For a reference copy of the document with all sections, see nature.com/documents/nr-reporting-summary-flat.pdf

Life sciences study design

All studies must disclose on these points even when the disclosure is negative.

Sample size	N/A
Data exclusions	N/A
Replication	N/A
Randomization	N/A
Blinding	N/A

Reporting for specific materials, systems and methods

We require information from authors about some types of materials, experimental systems and methods used in many studies. Here, indicate whether each material, system or method listed is relevant to your study. If you are not sure if a list item applies to your research, read the appropriate section before selecting a response.

Materials & experimental systems

n/a	Involved in the study	
[x]	[ ]	Antibodies
[x]	[ ]	Eukaryotic cell lines
[x]	[ ]	Palaeontology and archaeology
[x]	[ ]	Animals and other organisms
[ ]	[x]	Human research participants
[x]	[ ]	Clinical data
[x]	[ ]	Dual use research of concern

Methods

n/a	Involved in the study	
[x]	[ ]	ChIP-seq
[x]	[ ]	Flow cytometry
[x]	[ ]	MRI-based neuroimaging

Human research participants

Policy information about studies involving human research participants

Population characteristics	The manuscript is about a case report of 74-year-old man who received Personalized bacteriophage therapy to treat pandrug-resistant spinal P. aeruginosa infection
Recruitment	The patient was crecruited in a French referral center for the management of complex bone and joint infection. Our center is developing phage therapy as a treatement option throughout a dedicated program
Ethics oversight	Local ethics committee approved the management of this patient

Note that full information on the approval of the study protocol must also be provided in the manuscript.
